# Preparation and Study of Antifouling and Fouling-Release Surface Materials from Copolymers with Anchoring Functional Groups

**DOI:** 10.3390/ma16237367

**Published:** 2023-11-27

**Authors:** Fei Wan, Wenwen Yan, Chao Feng, Ruixuan Tong, Linlin Zhang

**Affiliations:** School of Civil Engineering, Qingdao University of Technology, Qingdao 266033, China; shuoyiecool@sina.com (F.W.); yanwenwen_2009@163.com (W.Y.); a19862223407@163.com (R.T.); 17863953125@163.com (L.Z.)

**Keywords:** antifouling, fouling-release property, catechol, copolymer

## Abstract

Marine biofouling is a worldwide problem in marine systems. Nowadays, innovative non-toxic antifouling and fouling-release materials are highly desirable. In this study, a strategy for preparing antifouling and fouling-release materials via one-step dip coating is reported. Copolymers were synthesized via the polymerization of a monomer with catechol sticky functional groups and four monomers with antifouling- or fouling-release functional groups, respectively. The copolymers could assemble onto different material surfaces, such as metals and plastics, using biomimetic catechol groups via multivalent complex bonding. The catechol groups were helpful for adhesion onto the surfaces, while the other functional groups endowed the coatings with antifouling or fouling-release properties. The effects of modifying the substrates using these copolymer coatings were verified via X-ray photoelectron spectroscopy; images of *Chlorella* cell and *Ulva* zoospore settlement were taken using a microscope and scanning electron microscope. The copolymer-coated surfaces, especially the surface modified by DOPA–PSPMA, displayed the best antifouling activity, and surface modification via DOPA–PTMETH was shown to be the most effective for producing the fouling-release property in the settlement assay.

## 1. Introduction

Biofouling presents micro- and macroorganisms or their excretion products on surfaces, which has become a widespread problem for marine vessels and infrastructure [1,2,3]. Many traditional effective antifouling methods, such as toxic-release coatings, have been restricted by legislation because of their significant adverse environmental effects [4]. Therefore, innovative non-toxic antifouling materials are highly desirable [5,6,7,8,9,10].

Surface chemical modification is one of the key approaches for endowing marine vessels with antifouling properties. Many materials with universal antifouling functions have been screened out [10,11,12,13,14]. Antifouling surfaces prepared via grafting polymers from initiators or grafting polymers with functional groups have been reported [8,10,15,16,17]. Chilkoti et al. prepared poly-(OEGMA) using SI–ATRP to resist protein adhesion [18]. Jiang et al. discussed antimicrobial cationic surfaces that can effectively kill bacterial [19]. Researchers have found that hydrophilic polymers such as poly(ethylene glycol) and zwitterionic polymers have significant antifouling properties [20,21,22,23]. Copolymers were also regularly reported for antifouling applications [14,24]. Liu et al. reported the synthesis of zwitterionic–phosphonic copolymers for the efficient antifouling surface settlement of diverse biomedical metals [25]. Li et al. reported that a polyether–thiourea–siloxane copolymer was synthesized by introducing thiourea and some ether groups into the MQ silicone resin polymer, which showed the most effective antifouling ability, via free radical polymerization [26]. Guo et al. designed a new surface-active copolymer based on hydrophilic polyvinylpyrrolidone and PDMS and then incorporated it into a crosslinked PDMS matrix to create a surface-renewable antifouling coating [27]. Xu et al. used an innovative photopolymerization technique to develop sulfur-containing polymer-grafted antifouling surfaces [28].

Although these approaches have been shown to be effective for modifying polymers onto material surfaces, specific interactions between interfacial modifiers (such as thiolate on noble metals and organosilane and polyamines on oxides) are also required, which are laborious and costly and cannot be easily used in practical applications [29]. Effective and simple one-step methods are strongly required. Catechol is the side chain of DOPA, which has widely been found in mussel adhesive proteins. It has been shown to be very important in surface-independent anchor molecules [30,31,32]. Many studies have shown that catechol groups can form covalent bonds and hydrogen bonds, and they also had strong physical interactions with material surfaces in [33]. The high concentration of DOPA at the adhesive interface showed the possible mechanism of enhancing adhesion. This specific study has stimulated significant interest in exploiting catechol for enhancing the interfacial adhesion of material surfaces. Polymers with catechol groups showed strong interfacial adhesion strength on many material surfaces, especially on wet surfaces, in [34]. Jiang et al. reported an antifouling polyampholyte polymer with catechol groups that was formed polymer coating and prevented protein adsorption [35]. Xiong et al. synthesized copolymers with different topologies, antifouling blocks, and terminal poly(dopamine-acrylamide) anchoring blocks and also used them for antiprotein absorption, antibacterial settlement, and antimarine fouling [36]. Park et al. presented highly antibiofouling multiloop polyethers functionalized with mussel–biomimic catechol groups with varying loop dimensions [37]. Sathyan et al. reported the synthesis of zwitterionic copolymers and their ability to form antifouling coatings on porous hydroxyapatite (used as a mimic of dental coatings) [38]. Yeon et al. synthesized a zwitterionic dopamine derivative containing catechol and amine groups and also demonstrated its excellent antifouling material surface by showing how it could control the oxidation of ZW–DOPA [39].

In this study, four kinds of copolymers containing antifouling pendant side groups (including hydrophilic groups, cationic surfactant functional groups, anionic surfactant functional groups, and hydrophobic groups) and catechol adhesive groups via polymerization were obtained. Settlement tests with *Chlorella* cells and *Ulva* zoospores were carried out to investigate the antifouling and fouling-release properties of the copolymer-modified material surfaces.

## 2. Materials and Methods

### 2.1. Materials and Characterization

3-Hydroxytyramine hydrochloride, α,α′-Azobisisobutyronitrile (AIBN), 3-sulfopropyl methacrylate potassium salt (SPMA(K)), 3,3,4,4,5,5,6,6,7,7,8,8,8-Tridecafluorooctyl methacrylate (TMETH), 2-(methacryloyloxy) ethyl-trimethylammonium chloride (METAC), and oligoethylene glycol monomethyl ether methacrylate (OEGMA, average Mn 500, 50 wt. % in H_2_O) were obtained from Sigma-Aldrich (St. Louis, MO, USA). Methacryloyl chloride was obtained from Alfa Aesar. Chemical reagents of Na_2_B_4_O_7_·10H_2_O and Na_2_CO_3_·H_2_O were obtained from Chinese Tianjin Chemical Reagents Corp. (Tianjin, China).

*Chlorella* cells and *Ulva* zoospores were obtained from Marine Biology Institute of Shandong Province (Jinan, China), and their suspensions were prepared using the standard method.

Information on the chemical compositions of the surfaces of the copolymer-modified substrates were characterized via XPS (PHI-5702, Phisical Electronics Corporation, Chanhassen, MN, USA) using Al KR radiation. The molecular weights of the copolymers were determined using GPC (Dawn Heleos System, Wyatt Technology, Santa Barbara, CA, USA) in water and DMF. The molecular weights were determined relative to the narrow molecular weight polystyrene standard. Optical images of cell and zoospore settlement on the surfaces the substrates were obtained using a microscope (BX51, Olympus Corporation, Tokyo, Japan). Micrographs of cells and zoospores were taken via SEM (JSM-5600LV, JEOL Ltd., Tokyo, Japan). All the samples were treated with 2.5% glutaraldehyde before being characterized via SEM. Coating thickness was measured using spectroscopic ellipsometry (UVISEL, Horiba Jobin Yvon, Montpellier, France) using a He-Ne laser at a fixed angle of 70°. Ten measurements were recorded per sample.

### 2.2. The Synthetic Method of Monomer

**Scheme 1:**
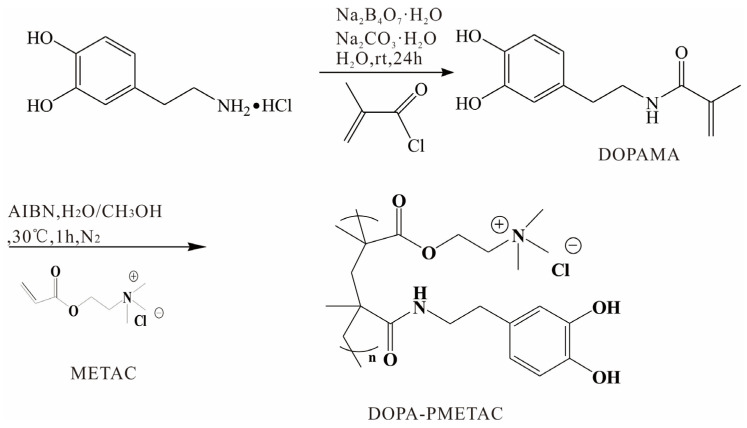
Synthesis of the PDOPA–PMETAC copolymer.

*N*-(3,4-dihydroxyphenyl)ethyl methacrylamide (DOPAMA) was prepared as shown in Scheme 1 [40]. Next, 3.83 g Na_2_B_4_O_7_·10H_2_O and 100 mL water were added into a 250 mL round-bottomed flask. Subsequently, 1.9 g dopamine·HCl was added after the solution had been degassed in an Ar atmosphere for more than 15 min, and the mixture was stirred for 10 min; this was followed by adjusting pH to 10 using Na_2_CO_3_·H_2_O.

The solution was cooled in an ice-water bath, and 1.05 g of methacryloyl chloride was added dropwise. The reaction solution was stirred for 24 h under an Ar atmosphere at 25 °C and then acidified to pH 2 using 6 M hydrochloric acid aqueous solution before being extracted using 100 mL ethyl acetate three times. The organic extracts were dried using anhydrous Na_2_SO_4_, and the brown solid was obtained via distillation under reduced pressure. The pure solid DOPAMA was then obtained using silica gel column chromatography.

### 2.3. Polymerization Methods

As shown in Scheme 1, PDOPA–PMETAC was synthetized via the polymerization reaction of METAC solution and DOPAMA. Briefly, 8 mL METAC solution, 44 mg DOPAMA, and 7.2 mg AIBN were all dissolved in 5 mL pure water and 5 mL methanol. Then, the mixed solution was charged using N_2_ for 10 min and kept in a N_2_ atmosphere for 1 h via stirring at 30 °C. The precipitation was obtained in methanol. The PDOPA–PMETAC copolymer was washed with methanol three times and then dried in a vacuum oven for 24 h.

PDOPA–POEGMA was synthetized via the precipitation polymerization reaction of the OEGMA solution and DOPAMA. Briefly, 5 mL OEGMA, 44 mg DOPAMA, and 7.2 mg AIBN were dissolved in 5 mL methanol. Then, the mixed solution was charged using N_2_ for 10 min and kept in a N_2_ atmosphere for 1.5 h via stirring at 35 °C. The PDOPA–POEGMA copolymer was washed with methanol three times and then dried in a vacuum oven for 24 h.

PDOPA–PSPMA was synthetized via the polymerization reaction of SPMA(K) powder and DOPAMA. Briefly, 0.96 g SPMA(K), 22 mg DOPAMA, and 7.2 mg AIBN were dissolved in 6 mL pure water and 4mL methanol. Then, the mixed solution was charged using N_2_ for 10 min and kept in a N_2_ atmosphere for 1 h via stirring at 30 °C. The precipitation was obtained in methanol. The PDOPA–PSPMA copolymer was washed with methanol three times and then dried in a vacuum oven for 24 h.

PDOPA–PTMETH was synthetized via the polymerization of TMETH liquid and DOPAMA. Briefly, 0.86 g TMETH, 88 mg DOPAMA, and 7.2 mg AIBN were dissolved in 20 mL methanol. Then, the mixed solution was charged using N_2_ for 10 min and kept in a N_2_ atmosphere for 5 h via stirring at 45 °C. The obtained PDOPA–PMETAC copolymer was washed with methanol three times and then dried in a vacuum oven for 24 h.

The number average molecular weight and molecular weight distribution values of the synthetized copolymers are shown in Table 1.

**Table 1:** Number average molecular weight and molecular weight distribution values of synthetized copolymers.

Copolymer	*M*_n_ (g mol^−1^)	*D*
PDOPA–PMETAC	7900	1.17
PDOPA–POEGMA	9800	1.21
PDOPA–PSPMA	7200	1.12
PDOPA–PTMETH	5600	1.28

### 2.4. Surface Modification by Copolymer Coatings on Substrates

Si, Cu, Fe, Ti, and PTFE wafers were all cleaned in pure methanol via sonication for 10 min. Then, all the wafers were immersed in 20 mg/mL copolymer solution for 12 h at 25 °C. After that, all the coating modified wafers were rinsed in water and methanol, respectively, and then dried in a vacuum oven.

### 2.5. Settlement and Adhesion Tests of Cells and Zoospores

All the modified silicon wafer samples were immersed in distilled water for 12 h and then transferred to artificial seawater for 1 h for the settlement and adhesion tests. All these samples were left on glass containers individually, and then the containers were filled with 20 mL 1.1 × 10^6^ cells mL^−1^ *Chlorella* suspension and 7.8 × 10^5^ spores mL^−1^ *Ulva* zoospores. All the samples were kept in *Chlorella* cell and *Ulva* zoospore suspensions for 3 h and then rinsed in new artificial seawater to remove unattached cells or spores. The cells or zoospores were counted based on observing 30 random fields of the 5 replicates for each substrate sample. All the removal rate data were obtained by exposing these substrate samples to 35 kPa water impact pressure and calculated from the settlement cells and zoospores density between these substrate samples before and after exposure to water.

## 3. Results and Discussion

### 3.1. Chemical Compositions of the Surfaces

**Figure 1:**
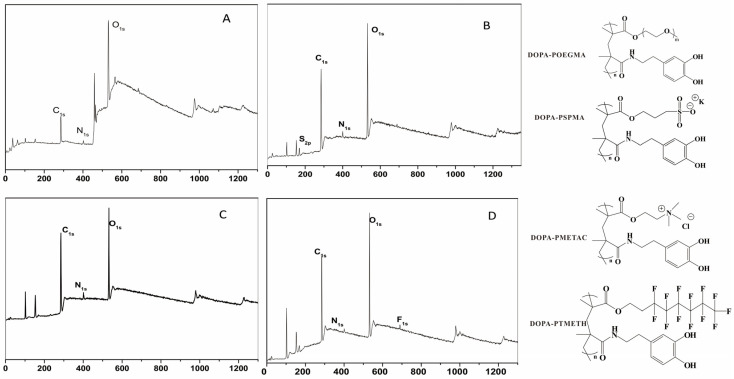
The XPS spectra of the (**A**) PDOPA–POEGMA, (**B**) PDOPA–PSPMA, (**C**) PDOPA–PMETAC, and (**D**) PDOPA–PTMETH coatings.

Surface modification on Si wafer substrates by four copolymers was characterized via XPS. Figure 1 illustrates that the XPS spectra of PDOPA–POEGMA-, PDOPA–PMETAC-, PDOPA–PSPMA-, and PDOPA–PTMETH-modified substrates showed the presence of C, O, N elements. The XPS spectrum of the PDOPA–PSPMA coating exhibited the S_2p_ signal at 162.5 eV, originating from the C-S bond; the XPS spectrum of the PDOPA-PTMETH-modified substrates exhibited the F_1s_ signal at 689.1 eV from the C-F covalent bond. Ellipsometry was used to measures the dry thickness of the PDOPA–POEGMA, PDOPA–PMETAC, PDOPA–PSPMA, and PDOPA–PTMETH coatings on the Si wafers after being immersed in 20 mg/mL copolymer solution for 12 h at 25 °C, and the values were 7.48 ± 2.27 nm, 5.38 ± 1.86 nm, 4.63 ± 1.55 nm, and 3.79 ± 1.93 nm, respectively. It is clear that all these substrate surfaces had been successfully modified by these copolymer coatings. The authors of [32,33] indicated that the catechol side chains could anchor the polymers onto surfaces, and with this and the results from Figure 1 in mind, the functional groups of POEGMA, PSPMA, PMETAC, and PTMETH were proved to be on the surfaces of the PDOPA–POEGMA, PDOPA–PMETAC, PDOPA–PSPMA, and PDOPA–PTMETH coatings, respectively.

**Figure 2:**
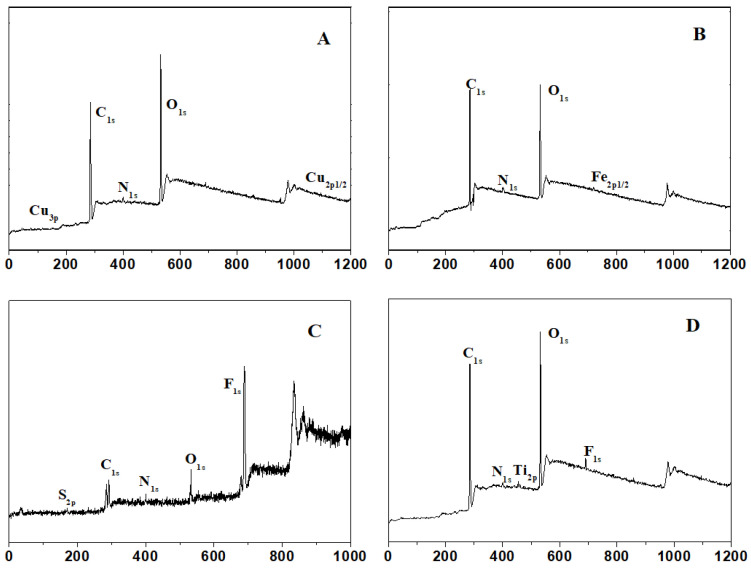
The XPS spectra of (**A**) a PDOPA–POEGMA-modified Cu substrate, (**B**) a PDOPA–PMETAC-modified Fe substrate, (**C**) a PDOPA–PSPMA-modified PTFE substrate, and (**D**) a PDOPA–PTMETH-modified Ti substrate.

Figure 2 shows the XPS spectra of the copolymer coatings on the Cu wafer substrate, Fe wafer substrate, Ti wafer substrate, and PTFE wafer substrate. Surface modification was proved by the S_2p_, N_1s_, and F_1s_ signals. Adhesion onto metal and plastic substrates was also provided by the catechol groups. It is shown that the copolymers could be easily used on many material substrate surfaces, containing general marine artificial materials.

### 3.2. Antifouling and Fouling-Release Performances

Table 2 shows the relationship between the reduced settlement ratios and growth removal percentages of the cells/spores with the molar ratios of the DOPAMA and OEGMA/SPMA(K)/METAC/TMETH monomers. It is obvious that the copolymers synthetized by 1:20 of DOPAMA and OEGMA, 1:30 of DOPAMA and SPMA(K), 1:20 of DOPAMA and METAC, and 1:20 of DOPAMA and TMETH monomers showed the best antifouling and fouling-release properties. In these copolymers, the catechol groups from the DOPAMA monomer provided strong interfacial adhesion strength to the surfaces, the hydrophilic groups, cationic surfactant functional groups, and anionic surfactant functional groups, and the hydrophobic groups of the DOPAMA and OEGMA/SPMA(K)/METAC/TMETH monomers provided antifouling and fouling-release properties. Dense copolymer coatings should have appropriate quantities of catechol groups in order to be strongly modified on the substrate and provide excellent antifouling and fouling-release performances.

**Figure 3:**
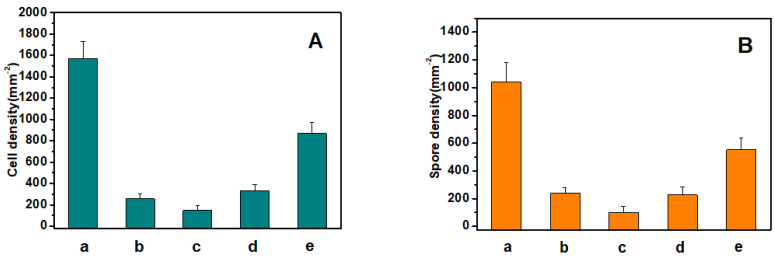
(**A**) *Chlorella* cells. (**B**) Settlement density values of *Ulva* zoospores on (**a**) the Si wafer substrate, (**b**) the PDOPA–POEGMA–modified wafer substrate, (**c**) the PDOPA–PSPMA–modified wafer substrate, (**d**) the PDOPA–PMETAC–modified wafer substrate, and (**e**) the PDOPA–PTMETH–modified wafer substrate.

*Chlorella* cell and *Ulva* zoospore settlement tests were used to verify the antifouling properties of all of the substrates. *Chlorella* have no flagella; they settle by and adhere to the substrate surface via proteins. *Ulva* zoospores have flagella to swim onto the proper surface and then settle [6]. The results of the settlement test on the Si wafer substrate surface and the copolymer-modified substrate surfaces are shown in Figure 3. It is shown that compared with the control Si wafer substrate, the DOPA–POEGMA-, DOPA–PMETAC-, DOPA–PSPMA-, and DOPA–PTMETH-modified substrates can significantly inhibit the settlement of *Chlorella* cells and *Ulva* zoospores, which could reduce 83 ± 3%, 91 ± 3%, 78 ± 3%, 45 ± 3% *Chlorella* cell settlement, respectively, and 76 ± 4%, 89 ± 3%, 77 ± 3%, 48 ± 4% *Ulva* zoospore settlement, respectively. The density values of the *Chlorella* cells (147 cells mm^−2^) and *Ulva* zoospores (101 spores mm^−2^) of the DOPA–PSPMA-modified substrate was the lowest, which means that the DOPA–PSPMA-modified substrate was the most effective in protecting against cell and spore settlement, while the DOPA–PTMETH-modified substrate was worse than all of the other substrates. Overall, the modifying copolymer coatings can efficiently inhibit cell and zoospore settlement and adhesion.

**Figure 4:**
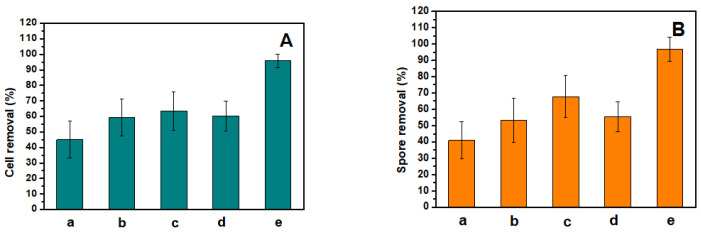
The removal percentages of (**a**) the Si wafer substrate, (**b**) the PDOPA-POEGMA-modified wafer substrate, (**c**) the PDOPA–PSPMA-modified wafer substrate, (**d**) the PDOPA-PMETAC-modified wafer substrate, and (**e**) the PDOPA-PTMETH-modified wafer substrate after the settlement of (**A**) *Chlorella* cells and (**B**) *Ulva* zoospores.

Figure 4 shows the cell and spore removal percentages of different substrates via water scouring. It is shown that compared with the control Si wafer substrate, the PDOPA–POEGMA-, PDOPA–PMETAC-, PDOPA–PSPMA-, and PDOPA–PTMETH-modified substrates could enhance the removal of *Chlorella* cells and *Ulva* zoospores much more efficiently, improving the removal percentage of *Chlorella* cells by 14 ± 1%, 18 ± 3%, 15 ± 3%, 49 ± 2%, respectively, and improving the removal percentage of *Ulva* zoospores by 15 ± 2%, 17 ± 1%, 16 ± 2%, 50 ± 2%, respectively. The copolymer coatings allowed the adhesive cells and zoospores to be easily released. Because of the low surface energy of the F element, the PDOPA–PTMETH-modified substrate was the most effective in releasing cells and zoospores via water shear force; there were almost no cells or spores on this substrate’s surface after exposure to water scouring. Therefore, these copolymer coatings, especially the PDOPA–PTMETH coating, could strongly improve the fouling-release performance of material substrate surfaces.

**Figure 5:**
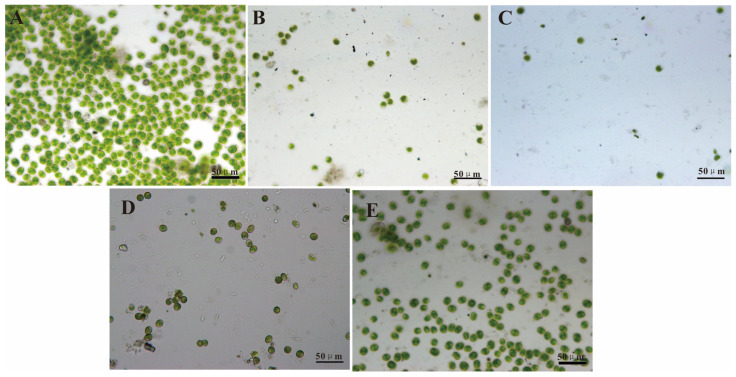
Images of *Chlorella* cells settled for 3 h on (**A**) the Si wafer substrate, (**B**) the PDOPA–POEGMA-modified substrate, (**C**) the PDOPA–PSPMA-modified substrate, (**D**) the PDOPA–PMETAC-modified substrate, and (**E**) the PDOPA–PTMETH-modified substrate.

**Figure 6:**
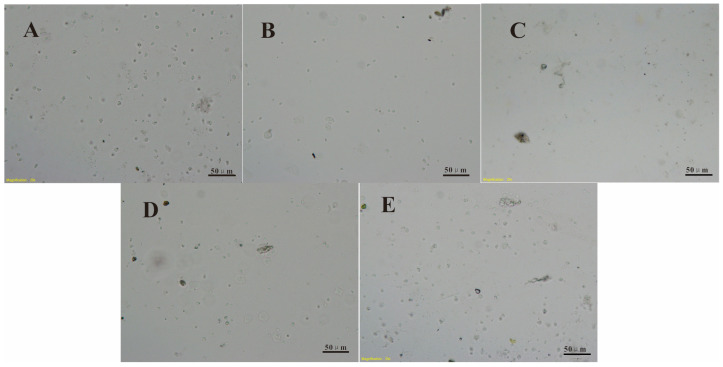
Images of *Ulva* zoospores settled for 3 h on (**A**) the Si wafer substrate, (**B**) the PDOPA–POEGMA-modified substrate, (**C**) the PDOPA–PSPMA-modified substrate, (**D**) the PDOPA–PMETAC-modified substrate, and (**E**) the PDOPA–PTMETH-modified substrate.

These findings are corroborated by the images in Figure 5 and Figure 6. The *Chlorella* cell and *Ulva* zoospore settlement densities on the DOPA–POEGMA-modified, DOPA–PMETAC-modified, DOPA–PSPMA-modified, and DOPA–PTMETH-modified substrate surfaces were much lower than those on the control Si wafer substrate surfaces (as shown in Figure 4A and Figure 5A). There were few single cells and zoospores on the PDOPA–PSPMA-modified substrate, and there were much more cell and zoospore colonies on the Si wafer substrate surface.

**Figure 7:**
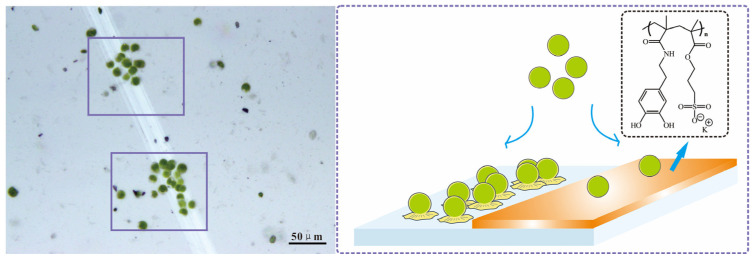
Image and schematic representation of *Chlorella* cells settled on a surface prone to biofouling and a substrate surface modified with the PDOPA–PSPMA coating.

The PDOPA–PSPMA-modified Si wafer substrate was scratched by tweezers to create a gap of about 20 μm without coating. After 3 h of *Chlorella* cell settlement, as shown in the images above, the only two colonies of *Chlorella* cells were both in the gap without the copolymer coating, while few individual cells could be observed on the coating area. The *Chlorella* cells have no flagella; they can only settle on surface through the action of gravity, adhere on the substrates via protein, and then continuously reproduce through cell division [6]. The schematic on the right of Figure 7 shows the reasoning behind the image on the left of the figure; if *Chlorella* cells settled in the gap area without PDOPA–PSPMA modification, they could easily adhere to the surface and reproduce through cell division along the gap to form cell colonies, while if *Chlorella* cells settled in the area modified with PDOPA–PSPMA, adhesion would be inhibited by the copolymer coating, meaning that they also could not reproduce.

**Figure 8:**
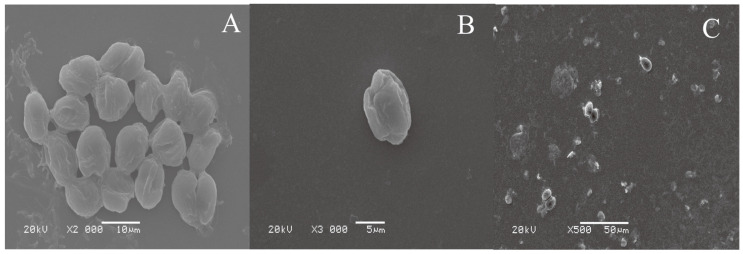
SEM images of *Chlorella* cells settled on (**A**) the Si wafer substrate, (**B**) the PDOPA–PSPMA-modified Si wafer substrate, and (**C**) the PDOPA–PTMETH-modified Si wafer substrate after water scouring (impact pressure of 35 kPa).

Representative SEM images of *Chlorella* cells settled on a surface prone to biofouling and a surface modified by the PDOPA–PSPMA coating are shown in Figure 8. In Figure 8A, the *Chlorella* cells have adhered onto the surface via the secretion of an adhesive glycoprotein [41] and created cell colonies; the cells were flat, and there were circles of blot around them. Figure 8B shows *Chlorella* cells on the PDOPA–PSPMA-modified substrate that have been separated from the substrate surface; no trace of a glycoprotein was present on the substrate surface. All these phenomena prove that these copolymer coatings, especially PDOPA–PSPMA, were very effective in inhibiting cell settlement. As shown in Figure 8C, after water scouring, there were few *Chlorella* cells on and clear glycoprotein traces on the PDOPA–PTMETH-modified substrate, which also proved that it had a significant fouling-release property.

## 4. Conclusions

For this paper, four kinds of copolymers containing catechol anchoring groups were prepared for preparing coatings with antibiofouling and fouling-release properties via a one-step procedure method. The catechol anchoring groups provided strong immobilization for the copolymer coatings onto the substrate surfaces, while the hydrophilic groups, cationic surfactant functional groups, anionic surfactant functional groups, and hydrophobic groups enhanced the antifouling and fouling-release properties. These copolymer coatings, especially the PDOPA–PSPMA coating, could be effective in inhibiting the settlement and adhesion of biofouling cells/spores; the PDOPA–PTMETH coating significantly enhanced the fouling-release property of the material’s substrate surface. These copolymer coatings can be used to modify the surfaces of various material substrates, as they contain general marine artificial materials such as metals and plastics. In conclusion, this new approach for easily producing materials with antifouling and fouling-release properties will be a very useful for practical applications.

## Figures and Tables

**Table 2:** Reduced settlement ratios and growth removal percentages of cells/spores of different samples.

Samples	Molar Ratio of Monomers	Reduced Settlement Ratio of Cells/Spores	Growth Removal Percentage of Cells/Spores
PDOPA–POEGMA	1:50	23 ± 5%/17 ± 6%	6 ± 1%/10 ± 2%
	1:40	41 ± 4%/38 ± 5%	11 ± 3%/13 ± 2%
	1:30	69 ± 4%/68 ± 3%	15 ± 2%/14 ± 1%
	1:20	83 ± 3%/76 ± 4%	14 ± 1%/15 ± 2%
	1:10	80 ± 5%/78 ± 3%	14 ± 3%/16 ± 3%
PDOPA–PSPMA	1:50	43 ± 2%/28 ± 4%	10 ± 2%/11 ± 1%
	1:40	72 ± 3%/65 ± 5%	11 ± 1%/13 ± 2%
	1:30	91 ± 3%/89 ± 3%	18 ± 3%/17 ± 1%
	1:20	88 ± 4%/75 ± 3%	18 ± 2%/15 ± 3%
	1:10	92 ± 4%/77 ± 2%	17 ± 1%/18 ± 3%
PDOPA–PMETAC	1:50	33 ± 3%/25 ± 5%	4 ± 1%/5 ± 2%
	1:40	42 ± 4%/37 ± 6%	11 ± 3%/13 ± 2%
	1:30	71 ± 4%/70 ± 3%	11 ± 2%/14 ± 1%
	1:20	78 ± 3%/77 ± 3%	15 ± 3%/16 ± 2%
	1:10	77 ± 2%/75 ± 4%	13 ± 2%/17 ± 2%
PDOPA–PTMETH	1:50	18 ± 1%/21 ± 2%	29 ± 3%/28 ± 2%
	1:40	26 ± 3%/25 ± 2%	35 ± 3%/41 ± 4%
	1:30	31 ± 4%/33 ± 3%	43 ± 1%/49 ± 2%
	1:20	37 ± 2%/41 ± 4%	47 ± 1%/50 ± 2%
	1:10	45 ± 3%/48 ± 4%	49 ± 2%/52 ± 1%

The samples are Si wafer substrates modified by copolymers synthetized by DOPAMA and OEGMA/SPMA(K)/METAC/TMETH monomers of different molar ratios.

## Data Availability

Data are contained in the article.
